# Prey Distribution, Physical Habitat Features, and Guild Traits Interact to Produce Contrasting Shorebird Assemblages among Foraging Patches

**DOI:** 10.1371/journal.pone.0052694

**Published:** 2012-12-20

**Authors:** Beth M. VanDusen, Stephen R. Fegley, Charles H. Peterson

**Affiliations:** 1 Institute of Marine Sciences, University of North Carolina at Chapel Hill, Morehead City, North Carolina, United States of America; 2 Curriculum for the Environment and Ecology, University of North Carolina at Chapel Hill, Chapel Hill, North Carolina, United States of America; Bangor University, United Kingdom

## Abstract

Worldwide declines in shorebird populations, driven largely by habitat loss and degradation, motivate environmental managers to preserve and restore the critical coastal habitats on which these birds depend. Effective habitat management requires an understanding of the factors that determine habitat use and value to shorebirds, extending from individuals to the entire community. While investigating the factors that influenced shorebird foraging distributions among neighboring intertidal sand flats, we built upon species-level understandings of individual-based, small-scale foraging decisions to develop more comprehensive guild- and community-level insights. We found that densities and community composition of foraging shorebirds varied substantially among elevations within some tidal flats and among five flats despite their proximity (all located within a 400-m stretch of natural, unmodified inlet shoreline). Non-dimensional multivariate analyses revealed that the changing composition of the shorebird community among flats and tidal elevations correlated significantly (ρ_s_ = 0.56) with the spatial structure of the benthic invertebrate prey community. Sediment grain-sizes affected shorebird community spatial patterns indirectly by influencing benthic macroinvertebrate community compositions. Furthermore, combining sediment and macroinvertebrate information produced a 27% increase in correlation (ρ_s_ = 0.71) with shorebird assemblage patterns over the correlation of the bird community with the macroinvertebrate community alone. Beyond its indirect effects acting through prey distributions, granulometry of the flats influenced shorebird foraging directly by modifying prey availability. Our study highlights the importance of habitat heterogeneity, showing that no single patch type was ideal for the entire shorebird community. Generally, shorebird density and diversity were greatest at lower elevations on flats when they became exposed; these areas are at risk from human intervention by inlet sand mining, construction of groins and jetties that divert sediments from flats, and installation of seawalls on inlet shorelines that induce erosion of flats.

## Introduction

Worldwide declines in shorebird populations, driven by coastal development and increasingly by direct and indirect effects of sea-level rise [1], [2], motivate environmental managers to better preserve, restore, create, and manipulate the critical coastal habitats on which these birds depend (e.g., [3]–[5]). Effective habitat management requires a better understanding of the factors determining habitat use and value to shorebirds. Study of foraging by shorebirds on various sand flat habitats is a long-standing subdiscipline of behavioral ecology (e.g., [6], [7]) that focuses primarily on whether patches of habitat are used non-randomly, and then on how birds discriminate among alternative patches of habitat to maximize their fitness.

The Ideal Free Distribution (IFD) optimal foraging model [8], in which the distribution of foragers reflects habitat suitability (based on factors such as prey density distribution and predation risk), has long provided a framework for studying patch choice decisions in foraging birds [9]–[11]. While foraging theory has helped to illuminate mechanisms that drive feeding patterns in targeted species by providing hypotheses, testing these hypotheses often requires use of simplified assumptions and a narrow focus on a single or limited number of shorebird species, prey species, and/or environmental variables. Because of these limitations, questions of how the entire community of shorebirds and its component guilds, defined by factors that influence foraging, are influenced by a broad array of environmental factors are more technically difficult to determine, but recent advances in multivariate statistical tools have opened the door for more comprehensive and powerful analyses of determinants of shorebird foraging.

Here, we ask whether the distribution of a community of foraging shorebirds conforms with expectations from the IFD; that is, whether the density distribution of foraging guilds matches the distributions of prey and presumed influential environmental variables. We build upon individual- and species-level understandings of foraging decisions in order to reveal more comprehensive guild- and community-level insights. Because the value of a habitat to foraging shorebirds can depend upon both local characteristics of the habitat patch and also the landscape-scale context in which that patch is located [12], we also consider how landscape characteristics affect habitat patch suitability. We apply powerful multivariate statistical approaches to test whether shorebird foraging guilds respond to differences in sedimentology and the benthic invertebrate prey community among a set of inlet intertidal sand flats (New River Inlet, North Carolina, USA). This site was selected because its spatial scale was ideal for a comprehensive community analysis: large enough to include multiple habitat patches of varying quality, yet small enough that foraging shorebirds could choose among those differing patches without traveling to distant locations.

## Materials and Methods

### Study Site

**Figure 1:**
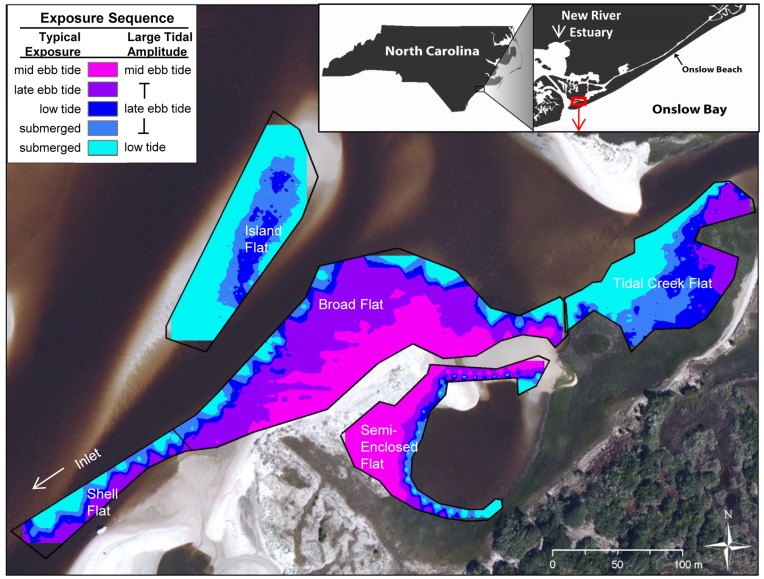
Location and elevation map of study site. (Note: apparent crenulations of the water edge on Broad and Semi-Enclosed Flats are artifacts of the elevation measurement technique.) Base layer photograph from Bing Maps Aerial Imagery.

Marine Corps Base Camp Lejeune is located on the North Carolina coast midway between Cape Lookout and Cape Fear. Onslow Beach, Camp Lejeune’s 12 km-long, southeast-facing barrier island, borders the Atlantic Ocean and is bounded on the southwest by the New River Inlet (Fig. 1). Our study site, comprised of five back-barrier intertidal sand flats, was located at the southwest tip of Onslow Beach and was managed by the base as a low impact/wildlife use zone. These sand flats experienced semi-diurnal tides with mean and spring tidal ranges of 1.3 and 2.0 m, respectively.

Flats ranged in area from about 0.5 to 2 ha at spring low tide, and were within 5–250 m of each other (Fig. 1). “Semi-Enclosed Flat” was the most sheltered of the group; it was bordered on the landward side by marsh and a sand spit and partially encircled a large pool of water that was connected to the estuary by a short, narrow (1–2 m wide) tidal channel. “Broad Flat” had the greatest intertidal area, and was located on the estuary-facing side of the sand spit. The southwestern tail of this flat was treated as a separate site and thus sampled separately because of obvious differences in surface shell cover (“Shell Flat”). “Island Flat” was a sandy shoal that emerged shortly before low tide near and parallel to Broad Flat. Located farthest from the ocean, “Tidal Creek Flat” bordered a marsh/tidal creek complex and was the muddiest of the sand flats. Broad Flat, Shell Flat, and Semi-Enclosed Flat emerged earliest in the tidal cycle, beginning about three hours before low tide. As the tide continued to ebb, Tidal Creek Flat was exposed next, followed by Island Flat. Tidal amplitudes were similar over the course of the study, although spring tides in mid December led to earlier exposure of Island Flat and greater exposed areas of all flats.

GPS location and elevation surveys were conducted using a Trimble Real Time Kinematic unit on 12 November 2008 and supplemented by additional measurements on 9 February 2009. Survey points were recorded at 0.5 m intervals along transects spaced approximately 10 m apart, perpendicular to the low-tide water line of each flat. In total, 4388 points were imported into ArcMap and inverse-distance weighted (IDW) to interpolate elevations for all exposed sand flat surfaces. IDW data were used to calculate surface area exposed for each flat at successive tidal heights (Table S1).

### Benthic Macrofaunal Invertebrates

**Figure 2:**
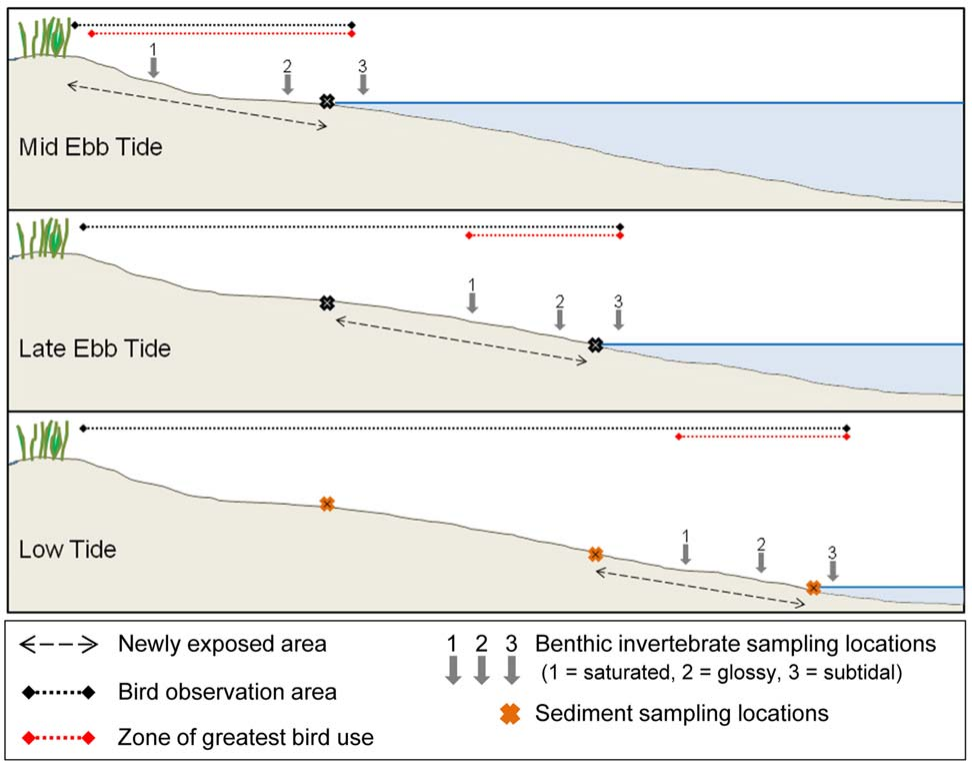
Sand flat sampling schematic. Sediment samples from all flats and all tidal heights were taken at low tide. The relative position of where the sediment samples would be taken subsequently is indicated by the darker crosses in each of the diagrams showing earlier tidal stages.

Benthic macroinvertebrates were sampled on 12 November 2008, approximately at the temporal mid-point of our shorebird censusing. We assumed benthic invertebrate densities did not exhibit dramatic variation from mid-October to mid-December, a period without major recruitment pulses (e.g., [13], [14]), major storms, or intense predation by fishes and crabs [15]. Flats were sampled for benthic macrofauna in a spatiotemporal pattern mimicking the general pattern of shorebird foraging at three stages in the tide and thus at three tidal levels: (1) three h before low tide (mid ebb tide) in the aerially exposed elevations; (2) 90 min before low tide (late ebb tide) in the newly exposed zone; and (3) low tide in the zone exposed last (Fig. 2). Because flats were sampled only if aerially exposed, not all flats could be sampled at all three times: Tidal Creek Flat was sampled first at late ebb and Island Flat only at low tide. The dynamic pattern of flat exposure on 12 November was typical of the study period (Table S1).

At each tidal stage, up to three microhabitats were distinguishable on each tidal flat, based on relative elevation and apparent water content: “saturated” (damp, but no apparent surface water), “glossy” (a film of surface water visible on the sediment), and “subtidal” (<3 cm of water cover). As sampling at later tidal stages focused on newly exposed areas, a “saturated” zone at late ebb tide was lower in elevation than a “subtidal” sample taken at mid ebb tide (Fig. 2). Because Island and Tidal Creek Flats had lower mean elevations than Broad, Shell, and Semi-Enclosed Flats, they contained only “glossy” and “subtidal” levels: no “saturated” samples were taken from these flats. A total of 7 replicate samples, each formed by the contents of a core 82 cm^2^ in surface area and 10 cm deep, was taken at each of the three microhabitat levels at each tidal stage on Semi-Enclosed Flat and at Broad Flat. Additionally, a full set of 3 replicate samples from each microhabitat was taken at Shell Flat; because this flat was much smaller than the others, fewer replicates were taken here in order to maintain comparable spacing between samples. Tidal Creek Flat was sampled at late ebb and low tides with 8 replicate samples per microhabitat level per tidal stage. A set of 7 replicate samples per level were obtained from Island Flat at low tide. The 10-cm depth was selected to capture benthic organisms that would be within probing range of the whimbrel (*Numenius phaeopus*), and to capture vertically-moving organisms that would periodically be available to surface-feeding shorebirds. Our sampling protocol was inclusive of all species of macroinvertebrates found in this depth range in order to match our focus on the entire community of shorebirds, which includes multiple feeding guilds. This depth was sufficient to capture most if not all organisms that serve as shorebird prey on intertidal sand flats of North Carolina inlets. The only deep-burrowing vermiform invertebrates in this biogeographic area (*Balanoglossus aurantiacus* and *Arenicola cristata*) were rare, judging from virtual absence of fecal casting evidence on the surface (BMV and SRF, pers. obs.). The only local deep-burrowing bivalve, the adult razor clam, *Tagelus plebeius*, can be recognized by its evident dual siphon holes at the surface [16], none of which were detected during our sampling. Although siphons of bivalves can contribute to some shorebird diets, the abundant bivalves of intertidal inlet flats in North Carolina (such as *Mercenaria mercenaria* and *Chione cancellata*) are all suspension feeders so, unlike some deposit feeders, their siphons are not extended and thus vulnerable when the intertidal habitat is exposed at low tide.

The 199 samples were returned to the laboratory on ice and sieved immediately on 0.5-mm mesh, with contents preserved using 10% buffered formalin with Rose Bengal stain. After sorting and enumerating by species, genus, or occasionally a higher taxonomic level, organisms were placed in 35% ethanol for ≤60 d until they could be dried at 60°C to constant mass (∼48 hours) and weighed. For each sample, dry mass was computed for the four major taxonomic groups: polychaetes; crustaceans (94% amphipods plus other small crustaceans such as isopods and larval crustaceans); bivalves; and gastropods.

One-way Model I ANOVAs were used to test each major group for differences in abundance, and separately in biomass, among flats at each tidal level. Both response variables were log (x+1)-transformed to meet statistical assumptions of normality and homogeneity of variance. To determine whether benthic community composition differed among flats, we performed one-way ANOSIMs (analysis of similarity; [17]) based on Bray-Curtis similarity matrices computed separately on abundance and biomass (log(x+1) transformed), with flat as the independent variable and individual samples as replicates. An n-MDS ordination based on similarity matrices of flat/tidal stage combination means, overlaid by results of a hierarchical cluster analysis (PRIMER v.6.1.11), was employed to depict groupings. We did not modify probabilities (α) to account for multiple testing (here or in any other analyses); rather, we chose to report unadjusted *P*-values but rely on them less than on *F* ratios, *R* statistics, and *t* statistics to assess potential biological patterns and provide evidence for rejection of null hypotheses.

### Sediments

Sediment samples were collected concurrently with benthic macrofauna. Each sediment sample consisted of three pooled 4.8-cm diameter cores taken to 10 cm depth. The three cores were taken haphazardly from the range of elevations sampled for benthos at each tidal stage, with specific placement blind to surface sedimentary characteristics. As the tide fell, mid ebb tide and late ebb tide waterlines on each flat were marked with flags. All sediment samples were taken at low tide, but replicate sets were taken based on each marked waterline to match the zones sampled for macrobenthos (Fig. 2). Consequently, Broad and Semi-Enclosed Flats had 7 replicate samples along the waterlines of each of the three tidal zones; Shell Flat had 3 replicates and Tidal Creek Flat had 8 replicates each from the late ebb tide and low tide waterlines; Island Flat, exposed only during low tide, had 4 replicate samples above the low-tide waterline and 4 below (<3 cm subtidally).

Each sediment sample was dried for 24 hr at 120°C in the laboratory and then weighed and sieved through a 2-mm mesh to remove the largest particles and calculate percent-gravel content. A ∼5 g sub-sample of each sand sample was processed through a CILAS laser particle size analyzer to determine particle size distribution. Grain sizes were binned into six groups based on the Udden-Wentworth scale (silt/clay: <63 µm, very fine sand: <125 µm, fine sand: <250 µm, medium sand: <500 µm, coarse/very coarse sand: <2000 µm, gravel: ≥2000 µm), and percent composition was computed for each sample. Grain-size group means were compared (1) among flats and (2) among tidal elevations within flats using one-way Model I ANOVAs.

To test for among-flat differences in grain-size distribution, we performed a one-way ANOSIM based on a Euclidean distance resemblance matrix with flat as the independent variable and grain-size distributions from individual samples as replicates. To remove collinearity, the size classes “coarse/very coarse sand” and “gravel” were excluded from the analysis because they were highly negatively correlated with “fine sand” (−0.898). We further constructed a similarity matrix from mean grain-size distributions for each flat/tidal stage combination and used it as the basis for an n-MDS ordination (Euclidean distance) and hierarchical cluster analysis.

### Shorebirds

Shorebird surveys were conducted on 14 dates between 15 October and 16 December 2008 (Table S1) by a single trained observer (BMV). Observations were conducted at 90-min intervals beginning three h before low tide (“mid ebb tide”), soon after Semi-Enclosed and Broad Flats first emerged, and ending at low tide when all flats were fully exposed. Each observation date was chosen to provide a falling tide during daylight. All observations were made on days without rainfall, with average wind speeds of 14 km hr**^−^** ^1^(range 0–32 km hr**^−^** ^1^) and air temperatures between 8 and 20°C (mean 14°C). During the first week of observations, several wooden stakes were inserted in Broad Flat in order to facilitate tidal height comparisons across dates. Shorebird surveys were conducted by walking the length of the sand spit along the vegetation line (Fig. 1) while counting and identifying all foraging shorebirds on each exposed flat. Because every flat was fully visible from this vantage point, double-counting of birds moving between flats was avoided. Few between-flat movements occurred during observation periods (which usually took about 10 min); if a bird did move between flats, its final location was the one recorded. Though they were not divided by water, Shell Flat and Broad Flat were observed separately because of differences in surface shell cover and human disturbance. To standardize human disturbance level, we excluded from analysis all Shell Flat bird counts that occurred while fishermen were on the flat. Observations were made using 8×40 porro prism binoculars at a minimum distance of ∼40 m from foraging birds. This distance was sufficient to avoid disturbing the birds.

To determine whether shorebird community composition consistently differed among flats over time, the PERMANOVA routine in PRIMER6 [18] was employed to analyze the shorebird community dataset using a randomized block design [19], with fixed factor “Flat” and random blocking factor “Date.” Each tidal stage was analyzed separately, and because Shell Flat was occupied by fishermen at times (and thus had a lower number of undisturbed replicates), it was excluded from this analysis. A dummy variable was added during the construction of resemblance matrices to prevent the loss of null samples (observations with no birds recorded) and associated degrees of freedom [20]. Before analysis, shorebird counts were standardized by area (birds per hectare).

To display patterns in the shorebird community, a non-metric multidimensional scaling (n-MDS) ordination using a Bray-Curtis similarity matrix was constructed from the means of each flat/tidal stage combination along with a hierarchical clustering (PRIMER v.6.1.11) of these means.

Significant multivariate PERMANOVAs were followed by univariate ANOVAs and Tukey-Kramer HSD post hoc contrasts to compare foraging densities at each tidal stage. Again, Shell Flat was excluded. In addition, we used χ^2^ to contrast foraging guild distributions, and computed Simpson’s D to compare shorebird community diversity among tidal stages.

### Integrated Analyses

The relationship between sediment grain-size distribution and benthic community structure was assessed using the BEST procedure in PRIMER6 [17]. BEST searches for high rank correlations between a fixed similarity matrix and resemblance matrices produced from a subset of possible explanatory variables that come from a second (“active”) similarity matrix. The degree to which the multivariate patterns of the fixed matrix match the patterns of the optimized subset matrix is the degree to which the subset variables “explain” the patterns in the fixed matrix. Our fixed matrix was the Bray-Curtis similarity matrix produced from the benthic abundance dataset. Because benthic and sediment samples were not matched one-to-one in the field, only “glossy” benthic samples were used in the first BEST analysis to provide the closest match to tide-line sediment samples. In a second analysis, all benthic samples were used. Both benthic and sediment datasets were reduced (by averaging replicates) to 12 matching composite samples– one per tidal stage exposed per flat. To remove collinearity in the sediment variables in both BEST analyses, “very coarse sand” and “gravel” were excluded.

The BEST procedure was also used to assess the spatiotemporal relationship between tidal flat sediment grain-size composition and the shorebird community. To match sediments with shorebird samples, the process of compositing sediment samples used in the sediment-benthos BEST analysis described above was repeated until a one-to-one sample correspondence was reached, providing each shorebird community sample with a matching sediment profile for a given flat at a given tidal stage. Shorebird observations from December were excluded because spring tides during that half month of sampling changed the distributions of birds on the flats relative to sediment sample locations, resulting in a poor spatial match. The composite shorebird density data were log(x+1)-transformed, and their Bray-Curtis similarity matrix served as the fixed matrix for the BEST analysis.

The relationship between benthic macrofaunal and shorebird communities was assessed using the BEST procedure as well. This analysis was performed using the shorebird density and benthic abundance datasets, with the similarity matrix from the shorebird dataset serving as the fixed matrix. To reduce numbers of benthic species (67), we included only those found in five percent or more of the total samples (Table S2). To ensure that the original benthic community patterns were preserved in this 14-species subset, we ran a BEST analysis (BVSTEP: [21]) using the complete benthic species list for the fixed matrix and the 14-species subset for the active matrix: the resulting high correlation (Spearman correlation coefficient ρ_S_ = 0.94) confirmed that benthic community patterns were preserved within the subset of most frequent species. We performed a BEST analysis using the fixed shorebird Bray-Curtis similarity matrix and the active benthic Bray-Curtis similarity matrix from the reduced species set. As with the sediment dataset in the previous analysis, benthic samples were composited until a one-to-one sample correspondence was reached between benthic and shorebird samples. In this way, each shorebird community sample was matched with the benthic community composition of a given flat at a given tidal stage.

A final BEST analysis drew upon all three datasets. The composited sediment and benthic macrofauna datasets were combined on a single spreadsheet to form an active matrix that supplied explanatory variables from both datasets at the same time. Once again, the Bray-Curtis similarity matrix from the shorebird dataset served as the fixed matrix for the BEST analysis.

### Ethics Statement

Marine Corps Base Camp Lejeune (MCBCL) Environmental Management Division approved this research, and MCBCL Range Control granted us access to the study site.

## Results

### Benthic Macrofaunal Invertebrates

Almost all (98%) invertebrates sampled belonged to one of four major taxonomic groups: polychaetes (53%), crustaceans (34%), bivalves (6%), or gastropods (5%) (see Tables S3, S4, S5, S6 for complete species list). Within the 16 families of polychaetes identified, 80% of individuals were either *Nereis* spp. (20%), *Capitella capitata* (17%), *Haploscoloplos robustus* (16%), *Heteromastus filiformis* (14%), *Paraonis* sp. (8%), or *Aricidea fragilis* (5%). Crustaceans consisted mostly of amphipods (94%), but also contained a few other small crustaceans including decapods (4% - mostly larval), and isopods. Bivalves were mainly *Donax variabilis*, *Gemma gemma*, or *Mercenaria mercenaria*, and the most abundant gastropods were *Nassarius obsoletus* and *Littorina irrorata*.

**Figure 3:**
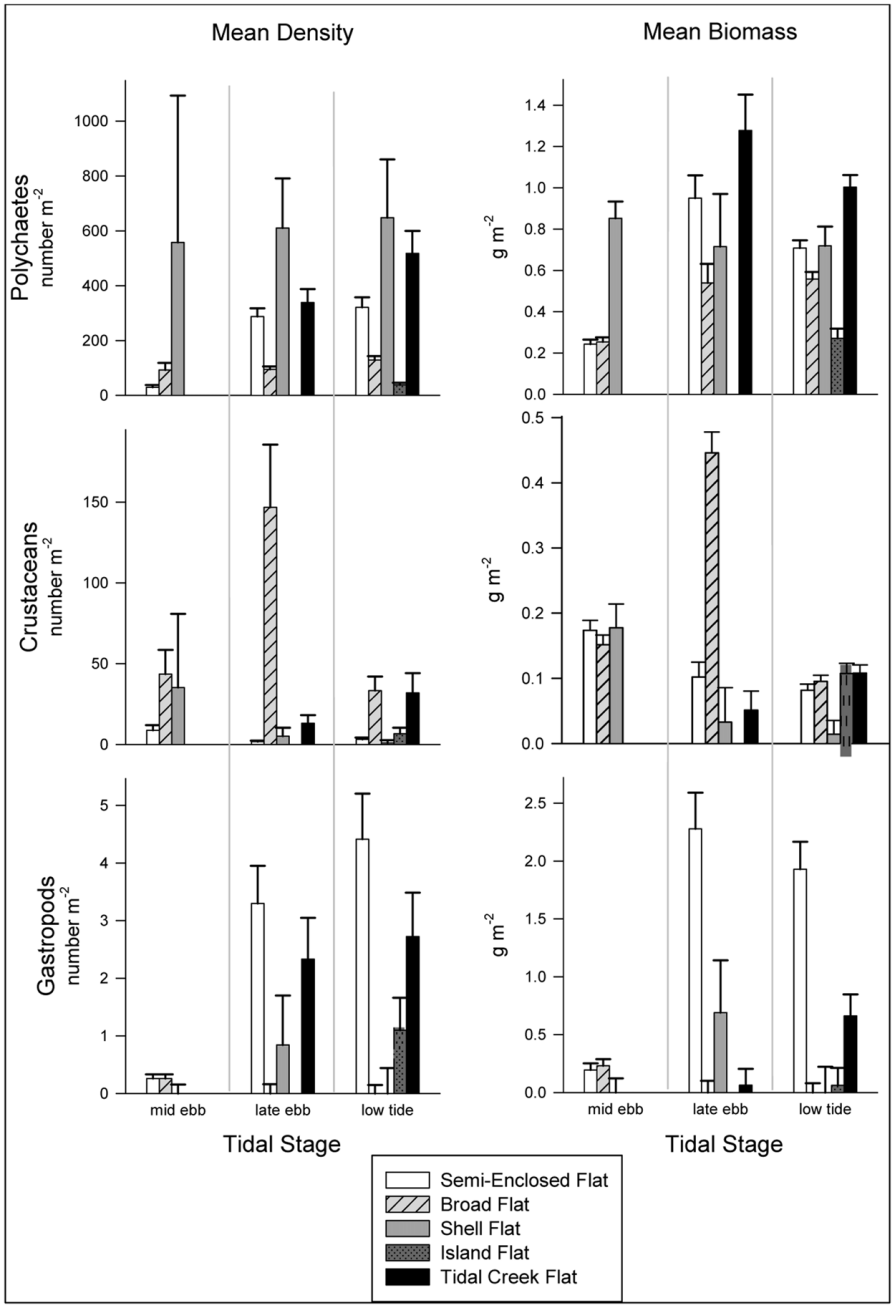
Mean density and biomass of major benthic macrofaunal taxa by flat and tidal stage. Bivalves are not shown because there was no significant difference in abundance among flats at any tidal stage, and only one significant difference in biomass (see Tables S7 and S8). Gastropod biomass includes shell mass.

One major difference among flats in benthic invertebrate density or biomass emerged from our analyses. Shell Flat had significantly higher polychaete densities than other flats at every tidal stage (Fig. 3, plus Table S7 for statistical test results). Polychaete biomass was also greater on Shell Flat than on Semi-Enclosed or Broad Flats at mid ebb tide, but did not differ significantly from other flats at late ebb tide or low tide (Table S8). Polychaetes represent the most abundant of the potential prey for shorebirds by a wide margin, with densities in the hundreds m**^−^** ^2^ compared to crustaceans in the tens m**^−^** ^2^ and gastropods and bivalves in the single digits m**^−^** ^2^ (Fig. 3). Crustacean density was significantly lower on Shell Flat than on Broad Flat at late ebb, but Shell Flat did not differ significantly from any other flat at any tidal stage in crustacean biomass or in either abundance or biomass of bivalves or gastropods (Fig. 3, Tables S7 & S8).

**Figure 4:**
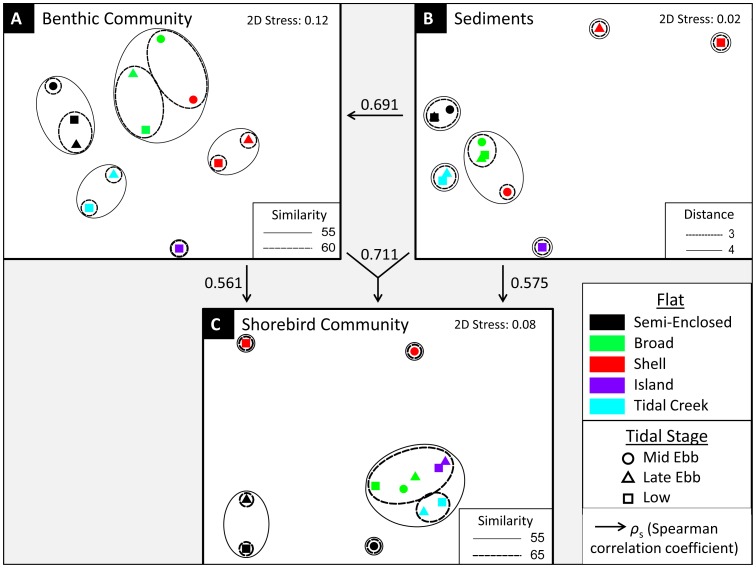
N-MDS ordinations displaying similarities/differences among shorebird and benthic community compositions and sediment grain-size distributions. Results of (A) benthic community composition, (B) sediment grain-size distribution, and (C) shorebird community composition n-MDS ordinations, comparing each combination of flat and tidal stage, with overlays of circles and ovals grouping flats according to cluster analysis results. Similarities given in percent. Grain-size cluster analysis overlay (B) is based on dissimilarity; increasing “Distance” values indicate an increase in dissimilarity among samples. Stress value of 0.12 (A) indicates that the 2-dimensional n-MDS ordination is an adequate and useful representation of sample relationships; stress value of 0.02 (B) indicates that the 2-dimensional n-MDS ordination provides an excellent representation of sample relationships; stress value of 0.08 (C) indicates a good representation. In (B), symbols for Semi-Enclosed Flat at late ebb and low tides are superimposed. In (C), Shell Flat at late ebb tide is not depicted because birds were never observed on the flat at that tidal stage.

In contrast, the benthic macrofaunal communities showed highly significant variability among all flats using abundances of the 14 most frequent species (ANOSIM, global R = 0.158, P<0.001). Individual pairwise comparisons of flats (Table S9) revealed significant differences between each flat pair except Semi-Enclosed and Shell Flat, and Semi-Enclosed and Tidal Creek Flat. N-MDS ordination and cluster analysis (Fig. 4A) showed substantial discrimination among flats based on their relative abundances of frequently occurring benthic macrofaunal species, although Shell Flat at mid ebb tide grouped with Broad Flat rather than with the later tidal stages on Shell Flat. Separation distances among clusters of points representing each flat did not differ greatly and showed no dramatic outlier (Fig. 4A). Results of an analogous ANOSIM applied to the composition of the macrobenthic communities based on biomass detected no significant difference among flats (global R = 0.003, P>0.05), nor did an n-MDS ordination of the community biomass dataset (not shown) reveal any pattern, an outcome common to biomass analyses because of the huge variability associated with inclusion of even a single large-sized specimen.

### Sediments

Each separate sediment grain size class revealed highly significant differences in percentage composition among tidal flats (ANOVA, P<0.001 for each size class). The flat most clearly distinguishable from all others was Shell Flat, which had the coarsest sediments by far (Table S10). Tidal Creek Flat was muddiest with a significantly higher percentage of silt and clay than any other flat, whereas Island Flat had a higher percentage of very fine sand, and Semi-Enclosed Flat had significantly more medium sand than any others (Table S10). Additional ANOVAs conducted to examine how sediment grain-size distributions varied with tidal level within the flat demonstrated invariant granulometry with elevation on Semi-Enclosed, Island, and Tidal Creek Flats. However, Broad Flat showed a progressive fining of sediments from mid ebb to low tide with significant increases in silt/clay percentages (ANOVA: F_2,18_ = 8.60, P<0.01) and a trend of increasing very fine sand. Shell Flat showed a marked coarsening of sediments from mid ebb to late ebb and low tide with increases in coarse/very coarse sand (ANOVA:F_2,6_ = 7.32, P = 0.02) and decreases from mid to late ebb and low tidal levels in fine sand (ANOVA: F_2,6_ = 13.32, P<0.01).

Results of analysis of similarity conducted on complete grain-size distributions of each tidal flat revealed a significant difference among the flats (ANOSIM: global R = 0.454, P<0.001). Subsequent n-MDS ordination of sediment grain size distributions by flat and tidal stage demonstrated that Shell Flat’s late ebb tide and low tide sediment composition differed considerably from all other flats and tidal stages (Fig. 4B), while the mid ebb tide sediments of Shell Flat clustered with sediments from Broad Flat (similar at all tidal stages). Semi-Enclosed Flat, Tidal Creek Flat, and Island Flat occupied unique positions in the two-dimensional ordination space (Fig. 4B), indicating unique sediment particle size compositions on each flat.

### Shorebirds

Shorebird community composition differed among the flats at each tidal stage, but exhibited no significant difference across the sampling dates (Table 1). All post hoc pairwise comparisons of tidal flats for late ebb and low tides demonstrated significant differences in shorebird community composition except for the contrast between Island and Tidal Creek Flats at late ebb tide (Table 1). Results of n-MDS ordination and cluster analysis conformed with the PERMANOVA results, showing clearly how the points depicting shorebird community composition in two dimensions segregated by flat (Fig. 4C). Broad and Island Flats were most similar to one another, whereas Semi-Enclosed and Shell Flats were both relatively distinct (Fig. 4C).

**Table 1:** Results of shorebird community PERMANOVA analyses testing for significance of variation among tidal flats.

			Tidal Stage		
Factors & Pairwise Comparisons		Statistic	Mid Ebb	Late Ebb	Low
Factor	FLAT	pseudo-*F*	7.702	17.635	17.827
		*P*	0.007	0.001	0.001
	DATE	pseudo-*F*	1.186	1.077	1.205
		*P*	0.347	0.374	0.252
	FLAT×DATE		excluded	excluded	excluded
	DF		1,7,15	3,7,26	3,12,47
Pairwise Comparisons	SE–BR	pseudo-*t*	2.775	4.761	4.900
		*P*	0.007	0.001	0.001
	SE–TC	pseudo-*t*	…	4.429	4.388
		*P*	…	0.003	0.001
	BR–TC	pseudo-*t*	…	4.996	3.368
		*P*	…	0.001	0.004
	IS–SE	pseudo-*t*	…	19.302	4.519
		*P*	…	0.003†	0.001†
	IS–BR	pseudo-*t*	…	4.685	3.852
		*P*	…	0.016†	0.001
	IS–TC	pseudo-*t*	…	4.184	2.997
		*P*	…	0.048†	0.001

PERMANOVA analyses used a randomized block design, and tidal stages were analyzed individually. Resulting *P*-values were obtained by permutation unless otherwise noted. Ellipses (…) indicate no data (flat pairs not exposed at that tidal stage). DF, degrees of freedom (Flat, Date, Total); BR, Broad Flat; IS, Island Flat; SE, Semi-Enclosed Flat; TC, Tidal Creek Flat.

† *P*-values were obtained using Monte Carlo sampling because low sample size did not yield enough possible permutations to get a reasonable test using the permutation method.

**Figure 5:**
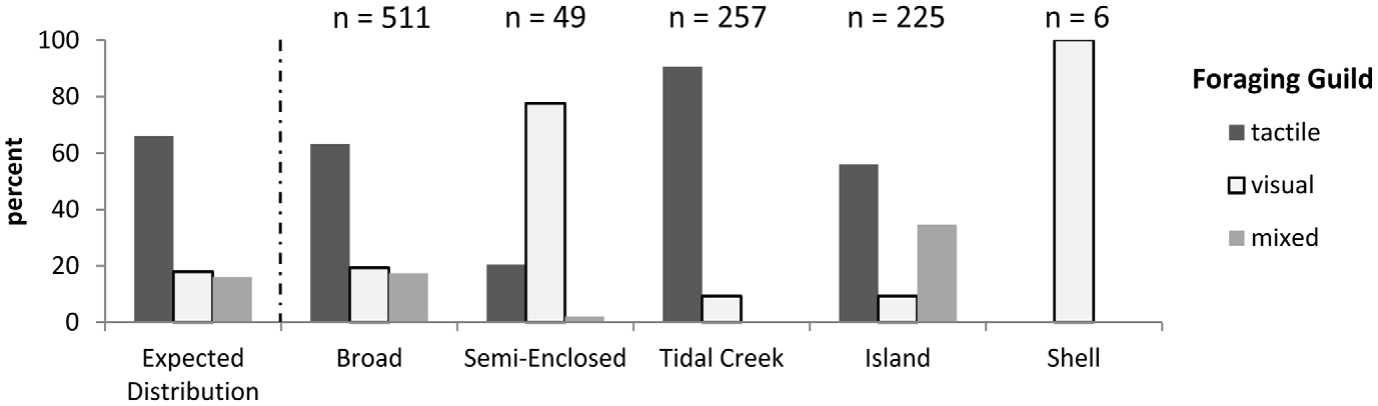
Flat use by shorebird foraging guild. Percentages are flat-specific. All tidal stages combined.

Similarly, an examination of flat use by shorebird foraging guild showed clear differences among flats (Fig. 5). When observations were pooled, tactile, visual, and mixed foragers comprised 66%, 18%, and 16% of total birds observed respectively. However, flat-specific foraging guild ratios differed from this overall distribution on all flats except Broad (Semi-Enclosed: χ^2^ (8) = 118.5; Tidal Creek: χ^2^ (8) = 75.4; Island: χ^2^ (8) = 61.5; Shell χ^2^ (8) = 27.4; P<0.001 for all). Unlike Broad Flat, Semi-Enclosed Flat had a large proportion (78%) of visual foragers, while Tidal Creek Flat was used almost exclusively (91%) by tactile foragers. Though Island Flat was not dominated by a particular foraging guild, it had a higher percentage of mixed foragers (35%) than the other flats. Shell Flat was rarely used by shorebirds, but the few birds that did use it were all visual foragers (3 species).

**Figure 6:**
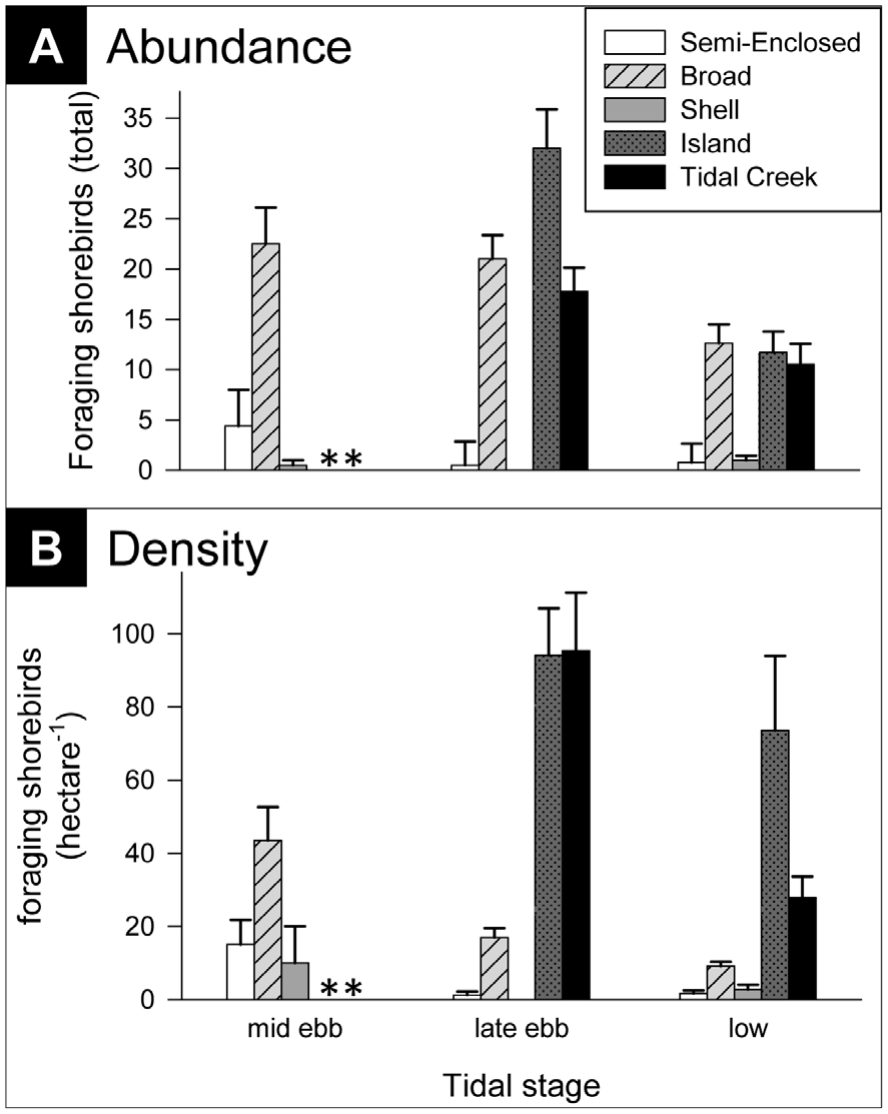
Mean foraging shorebird (A) abundances and (B) densities, by flat and tidal stage. Shell Flat is plotted for comparison only; it was not included in statistical tests due to low sample size when fisherman disturbance dates were excluded. Asterisks indicate submerged flats.

Univariate analyses of average shorebird density revealed many significant differences among flats (Table 2; we excluded Shell Flat from this analysis because of low sample size after eliminating fisherman-disturbance dates). At each of the three tidal stages, mean shorebird density on Semi-Enclosed Flat was consistently lower than any other flat with exposed surface at that tidal stage (Fig. 6). Semi-Enclosed Flat’s status as a low-density outlier was analogous to its departure in community composition from the other three flats analyzed (Fig. 4C).

**Table 2:** Results of one-way ANOVAs comparing mean shorebird densities among tidal flats, as a function of tidal stage.

Tidal Stage	*P*-value	*F* Ratio	DF	T-K post hoc contrasts
Mid Ebb	0.0247	6.32	1, 15	BR>SE†
Late Ebb	<0.001	24.37	3, 26	TC = IS>BR = SE
Low	<0.001	10.73	3, 47	IS>TC = BR = SE

BR, Broad Flat; IS, Island Flat; SE, Semi-Enclosed Flat; TC, Tidal Creek Flat. Shell Flat was not included in this analysis.

† No post hoc test necessary; inequality follows from ANOVA outcome.

**Figure 7:**
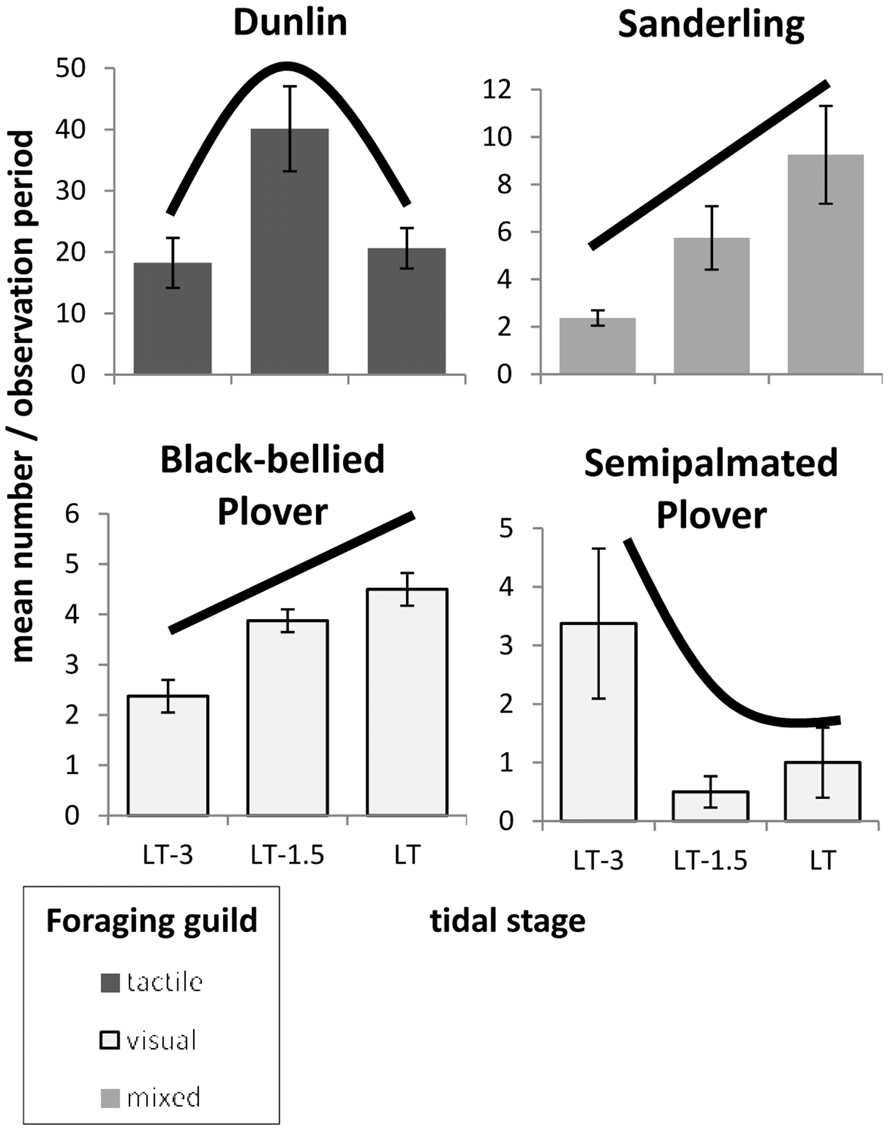
Trends in abundance of commonly observed shorebird species: by tidal stage. All flats combined.

The general pattern of change with ebbing tide revealed increasing total numbers of feeding shorebirds summed across all five tidal flats as the tide fell from its mid-ebb stage, as opposed to staying constant and simply becoming redistributed as new foraging areas become exposed and accessible (Fig. 6A). Additionally, the lower-elevation flats, Tidal Creek and Island, exhibited relatively dense concentrations of shorebirds, especially at late ebb (Fig. 6B). The peak in total abundance at late ebb tide was driven by the numerically-dominant dunlin (*Calidris alpina*), which was most abundant at that tidal stage (Fig. 7). Two other commonly observed species, sanderling (*Calidris alba*) and black-bellied plover (*Pluvialis squatarola*), tended to increase in abundance from mid ebb to low tide (Fig. 7). In contrast, the semipalmated plover (*Charadrius semipalmatus*) was most abundant at mid ebb tide, and rarely foraged on the flats at later tidal stages (Fig. 7). Rarely occurring species, including ruddy turnstones (*Arenaria interpres*), piping plovers (*Charadrius melodus*), willets (*Tringa semipalmata*), and yellowlegs (*Tringa* spp.) also contributed to observed species diversity. Though diversity decreased at late ebb tide with the influx of the numerically dominant dunlin (Simpson’s D = 2.0, 1.6, and 2.5 for mid ebb, late ebb, and low tide respectively), their low tide decline, combined with the increase in abundance of other common species (Fig. 7) and an increase in occurrence of rare species, resulted in an overall increase in shorebird community diversity at low tide.

### Integrated Analyses

A BEST analysis between sediment size classes and benthic macrofaunal community composition in the glossy zone (essentially the tide line) produced a Spearman correlation coefficient (ρ_s_) of only 0.119 with “silt/clay” and “very fine sand” size classes. However, the BEST analysis between sediments and composite benthic samples (from saturated, glossy, and subtidal samples) yielded a much higher optimized correlation (ρ_s_ = 0.691, p<0.01; with key contributing variables of “silt/clay”, “very fine sand”). The BEST analysis between composited sediment size distributions for each tidal elevation of each flat and the corresponding shorebird community composition based on average density at each tidal elevation of every flat had an optimized correlation of ρ_s_ = 0.575 (p<0.01) with 2 variables: “medium sand” and “fine sand” (the latter also standing as a proxy for “coarse/very coarse sand” and “gravel”). The BEST analysis comparing the composited samples of the 14-species subset of most frequent benthic invertebrates at each tidal elevation of every flat to the shorebird community composition based on abundances at each level of each flat optimized with 1 variable (crustaceans) at ρ_s_ = 0.561 (p<0.01). Finally, when all three composited datasets were used concurrently (correlating sediment size composition and benthic macrofaunal composition with shorebird community patterns), the BEST analysis optimized at ρ_s_ = 0.711 (p<0.01) with 4 variables (crustaceans, *Capitella capitata, Donax variabilis*, and “fine sand” (the equivalent of “coarse/very coarse sand” and “gravel” as well because of the strong collinearity among these three size classes in the composite dataset)).

We were unable to estimate or partition out effects of spatial autocorrelation for this study because we did not have a continuous gradient in flats across space that would facilitate those computations. However, spatial autocorrelation would most likely have reduced variation among flats across tidal stages, decreasing our ability to detect changes over time, thereby implying that patterns we demonstrate are conservatively estimated.

Visual examination comparing n-MDS plots A, B, and C (Fig. 4) documents the similarity in patterns among sediments, benthos, and the shorebird community. Fig. 4B shows that each flat is sedimentologically unique, with different flats forming different clusters. The single exception, Shell Flat at mid ebb tide (which clusters with Broad Flat), matches our observations because Shell Flat and Broad Flat were physically connected and Shell Flat did not have shelly sediments at its highest elevation. These patterns are echoed in the benthic community (Fig. 4A), where each flat has a unique composition of benthos except for Shell Flat at mid ebb tide, which is not distinguishable from the adjoining Broad Flat. The pattern displayed by the shorebird community (Fig. 4C) is similar to that of both the sediment and benthos, with Semi-Enclosed Flat and Tidal Creek Flat each clustering away from the others. Shell Flat, with its distinct sediments (Fig. 4B) and benthos (Fig. 4A), also separates from the other flats in bird community composition (Fig. 4C). In contrast, while Island Flat possesses distinct sediments and benthos (Fig. 4A & B), its shorebird community composition does not differ from that of Broad Flat (Fig. 4C).

## Discussion

### Shorebird Community Distribution Patterns

Our study demonstrates how a neighborhood of tidal flats found along a 400-m stretch of undeveloped, natural inlet shoreline can exhibit non-random spatial distributions of feeding shorebirds. Not all of these tidal flats, even those in close proximity, and not all elevations on a given flat are perceived or used equally by shorebirds. Both the total abundance of feeding shorebirds as well as the species composition varied across flats and dynamically as tidal elevation changed. As the tide fell from mid ebb to late ebb and then to low, certain changes in patterns of flat use by foraging shorebirds were observed repeatedly. Many foraging shorebirds like dunlin tended to move down in elevation as the tide fell, presumably taking advantage of the newly exposed, still wetted sediments where surface activity of benthic invertebrates may facilitate successful predation [22], [23]. Total shorebird abundance, summed across all flats, increased from mid ebb to lower tides, and diversity was highest at low tide. The flat at the lowest elevation, Island Flat, was especially heavily used once exposed at low tide, with shorebird densities greater than or equal to any other flat. In a study looking at the effects of the tidal cycle on shorebird habitat selection, Burger and colleagues [24] found that while shorebirds foraged on ocean and sound beaches shortly after high tide, as the tide fell and mudflats were exposed birds moved from the beaches to the mudflats. Similarly, two species that forage frequently on ocean-facing shores of Onslow Beach, the black bellied plover and sanderling, increased in abundance on the intertidal flats as the tide fell (Fig. 7). These tidally-linked foraging patterns led to increased shorebird diversity on the flats as low tide approached.

### Relating Shorebird Community Distribution to Benthos and Sediments

BEST analyses demonstrated that the shorebird community distribution correlated strongly with local variation in the benthic invertebrate community (ρ_s_ = 0.56), explaining a large portion of the pattern in shorebird foraging habitat use. Adding information on sediment grain-size distributions to the benthic invertebrate community dataset and forming a composite of predictor variables revealed that inclusion of grain size information further improved by about 27% the correlation with the distribution pattern of feeding shorebirds (ρ_s_ = 0.71). One might have expected the granulometry of the flats to have had its influence on where shorebirds feed indirectly via the strong impacts of grain size composition on the benthic invertebrates themselves. Indeed, grain size and benthic macrofauna exhibited a correlation coefficient of 0.69, indicating that much of the spatial variance in benthic invertebrate community composition could be explained by local changes in sediment size composition. Yet, the further direct explanatory value of adding in sediment grain size information to benthic invertebrate composition in explaining shorebird habitat use suggests the operation of direct effects of sediment size beyond indirect effects operating through determination of prey types and abundances (Fig. 4).

The strong relationship between benthic invertebrates as prey and feeding shorebirds as predators is predictable based on many earlier studies of feeding shorebirds (e.g., [25]–[31]). The direct contribution of sediment size to explaining shorebird feeding distributions after already accounting for its influence acting indirectly through benthic invertebrate prey seems likely to reflect the ability of some sediments to modify the availability of the benthic prey independent of their abundance [32]–[34]. This direct influence of sediment character may help explain a flat-specific anomaly in shorebird usage. Tidal Creek Flat was characterized by much higher silt/clay content than any other flat. In this study, densities of the tactile-foraging dunlin were much higher on Tidal Creek Flat than on Semi-Enclosed or Broad Flats, even though there were no significant differences in macroinvertebrate densities among the three flats. With its higher silt/clay percentages, sediments of Tidal Creek Flat were less porous, and its poor drainage resulted in the persistence of many small pools and areas covered with a thin veneer of water as the tide fell. Because these water-cover characteristics lead to prolonged surface activity in some macrobenthic prey organisms [22], [23], shorebirds that forage on these macrofauna likely experience increased prey availability under such conditions. Consequently, the higher foraging densities of dunlin on Tidal Creek Flat may have been a consequence of poor water drainage enhancing prey availability or detectability facilitated by higher prey activity [22], [35] or greater sediment penetrability for probing bills [36].

While shorebirds foraged heavily on Tidal Creek Flat, Shell Flat was used by very few birds despite its extraordinarily high polychaete densities (Fig. 3, Fig. 6). The sediments of Shell Flat contained the highest concentrations of gravel and coarse particles including shell fragments, which interfere with prey detection and capture by impeding sediment penetration by probing shorebirds [34]. Although substrate with a sizeable amount of coarse material may act as a refuge to infaunal prey [33], benthic invertebrates are still vulnerable to visually feeding shorebirds when at the surface [37]. Thus, while tactile, probing foragers experience a decrease in prey capture success, the guild of visual foragers appears better suited to utilize a coarse-sediment habitat. This seemed to be the case for Shell Flat, where the only species observed feeding were black-bellied plovers, piping plovers, and ruddy turnstones (see Table 3 for complete species list and associated foraging modes). Ruddy turnstones forage by flipping shells and coarse material and then looking for prey hidden underneath, a method perfectly suited for the sedimentary characteristics of Shell Flat. Black-bellied and piping plovers are both visual foragers that rely on surficial presence and activity of prey rather than substrate penetration to locate food items. In contrast, the visibly armored surface of Shell Flat apparently deterred shorebirds in the probing guild, such as the dunlin, which was never observed on Shell Flat while representing the dominant shorebird on all other flats (Fig. 5). Even with fisherman-disturbed dates removed, Shell Flat supported extremely low numbers of foraging shorebirds (0–2 birds per observation). The most parsimonious explanation for the enhanced abundance of polychaetes on Shell Flat is that armoring of the sediment surface inhibited dunlin and other probers, which served to provide polychaetes with a refuge against predation so their abundances were elevated above neighboring flats lacking shell armoring.

**Table 3:** Predominant foraging modes of all observed shorebird species.

Common name	Species	Predominant foraging mode
Ruddy turnstone	*Arenaria interpres*	visual
Sanderling	*Calidris alba*	mixed
Dunlin	*Calidris alpina*	tactile
Western sandpiper	*Calidris mauri*	mixed
Piping plover	*Charadrius melodus*	visual
Semipalmated plover	*Charadrius semipalmatus*	visual
Black-bellied plover	*Pluvialis squatarola*	visual
Willet	*Tringa semipalmata*	mixed
Yellowlegs	*Tringa* spp.	visual (in daylight)

### Landscape Influences

Another major distinction among flats in shorebird use, the comparatively low abundances of foraging shorebirds on Semi-Enclosed Flat (Fig. 6), cannot be explained by the distribution of prey: Semi-Enclosed Flat had comparable prey densities to the more heavily used flats. However, the surrounding habitat matrix may have contributed to the low foraging shorebird densities on Semi-Enclosed Flat at late ebb and low tides. The flat was nearly surrounded by marsh vegetation, so that as the flat area expanded with the falling tide its leading edge moved away from sparse vegetation on one side but approached dense marsh vegetation and tall trees on the opposite side of the small cove (Fig. 1). Vegetation proximity plays an important role in shorebird nest site selection [38], and may also influence choice of foraging site [39], [40]. While studying predation risk to small shorebirds, Dekker and Ydenberg [41] found that dunlin face an increased risk of predation by raptors as distance to vegetation decreases. In contrast to Semi-Enclosed Flat, the expanding edge of each of the other flats moved toward open water or another sand flat, providing increased distance from hidden predators or raptors hunting from perches.

While Semi-Enclosed Flat was used by few shorebirds, Island Flat experienced high densities of foraging birds when it was exposed at later tidal stages (Fig. 6B). The heavy usage of Island Flat was probably a consequence of its geography: as an island, it had roughly double the water-edge length of any equal-sized sand flat connecting to higher ground (Fig. 1). Edges play an important role for many species of foraging shorebirds including dunlin [24], [42], [43], the most abundant species on the New River Inlet sand flats at Onslow Beach. Dunlin and other “edge followers” (typically probers– see [43]) follow the moving tide line and exert heavy feeding pressure within that margin. Perhaps this intensity of foraging on Island Flat at low tide was responsible for the relatively low abundance of the two most important major taxa of prey invertebrates, polychaetes and crustaceans, which were both significantly higher on other flats. Consequently, the area-edge relationship may play a pivotal role in determining the foraging habitat value of a sand flat to foraging shorebirds.

Although much of the spatiotemporal variation in the composition of the community of foraging shorebirds can be explained by joint knowledge of the benthic macroinvertebrate community and sediment size composition, a considerable portion of the variability remains unexplained. Geographic components such as vegetation proximity and area-edge relationships likely account for some of this unexplained variability; however, we did not quantify the entire suite of potentially influential landscape variables in this study. Accordingly, we chose not to include limited landscape variables (alongside complete sets of prey species and sedimentary characteristics) in our analyses because their inclusion could compromise the interpretability of our results: any observed increase in variance explained by an included landscape variable might actually be driven by any number of additional unmeasured or co-varying variables.

### Behavior

It is possible that bird behavior also contributed to the unexplained variability in shorebird spatiotemporal distributions although the specific mechanisms are not clear. Agonistic behaviors did not play a major role in structuring patterns of patch use as few negative interactions were observed, consistent with species-specific literature (e.g., [44], [45]), which reports little to no territoriality or other agonistic behavior among non-breeding and/or wintering dunlin, semipalmated plover, or sanderling (North Carolina specific: [46]), and non-aggressive intraspecific spacing in black-bellied plovers [47]. Only once did we observe a bird being chased from a flat: a semipalmated plover chased a conspecific off of Semi-Enclosed Flat (the bird flew to Broad Flat). However, even spacing between individual feeding black-bellied plovers (>50 m) was evident. Although ruddy turnstones are known to interact aggressively with other shorebirds foraging in close proximity (<1 m) [48], we never observed any aggressive interactions between ruddy turnstones and other species on the flats.

### Conclusions

Prey abundance and availability (as mediated by sediment characteristics) explain much of the variation in foraging patterns of the shorebird community, a conclusion mirroring ones arising previously from studies of habitat selection by single species. Our multivariate analyses confirmed this relationship for guilds representing different foraging modes and revealed that no single patch type is ideal for all guilds that comprise the entire shorebird community. Habitat heterogeneity (defined by patches with varying sedimentological and elevational characteristics) is necessary to support the full spectrum of shorebirds with different prey preferences and foraging modes. Consequently, inlets containing extensive areas of heterogeneous intertidal sand and mud flats are important to sustaining functional diversity of shorebird communities.

Results of our study have important implications for shorebird conservation and coastal management. The sediments that form intertidal sand flats near inlets are part of a dynamic ocean beach and inlet sand-sharing system with a finite sand resource. When humans intervene in this sand-sharing system by erecting groins and jetties intended to capture and retain sand in front of a specific shoreline property to protect against erosion, such interventions disrupt the natural flow of sand and commonly result in downdrift sand deficits [49]–[51]. Similarly, seawalls constructed on inlet shores to protect shoreline development from wave damage and overwash function by redirecting the energy of breaking waves downward, which generates sediment erosion seaward of the walls and leads to loss of the intertidal habitat [49]–[51]. Finally, mining sands from ebb- or flood-tidal deltas within inlets during boat channel construction and maintenance or for use in beach nourishment removes sand from the littoral sand-sharing system [49], [51]. The changes induced by each of these engineering interventions impose a high risk of reduction in the volume of sand available for retention in extensive intertidal sand flats within inlets. Inlet flats are especially valuable foraging areas for many shorebirds because of their proximity to preferred nesting grounds near natural inlets, where frequent overwash inhibits development of dense vegetation and thereby sustains their high attractiveness to many shorebirds as nesting areas [52]. Consequently, shorebird conservation could be more effective if human access to inlet tidal flats was minimized during shorebird nesting and chick-rearing seasons in conjunction with current nesting-ground protection practices. Along with this seasonal management focus, preclusion of both engineered shoreline stabilization structures near inlets and inlet sand mining is critical to sustaining the heterogeneous intertidal feeding and nearby nesting habitats required by diverse shorebird assemblages.

## Supporting Information

Table S1 **Total intertidal area (hectares) of each tidal flat at the three tidal stages of observation, as a function of tidal amplitude.**
(DOCX)

Table S2 **Benthic macrofaunal species found in at least five percent of samples.**
(DOCX)

Table S3 **Phylum Polychaeta densities (organisms m^−2^) by flat.**
(DOCX)

Table S4 **Phylum Arthropoda densities (organisms m^−2^) by flat.**
(DOCX)

Table S5 **Phylum Mollusca densities (organisms m^−2^) by flat.**
(DOCX)

Table S6 **Flat-specific densities (organisms m^−2^) of organisms from additional phyla.**
(DOCX)

Table S7 **Results of one-way ANOVAs comparing benthic invertebrate abundance among tidal flats at each tidal stage, and post hoc contrasts using Tukey-Kramer HSD.**
(DOCX)

Table S8 **Results of one-way ANOVAs comparing benthic invertebrate biomass among tidal flats at each tidal stage, and post hoc contrasts using Tukey-Kramer HSD.**
(DOCX)

Table S9 **Results of post hoc pairwise comparisions of flats following Analysis of Similarity (ANOSIM) on benthic invertebrate community composition dataset of abundances by species (or genus, and in some cases higher taxon).**
(DOCX)

Table S10 **Results of Tukey-Kramer HSD post hoc comparisons of all possible pairs of flats based on percentage composition of each size class of sediments.**
(DOCX)
