# Supplementary material for: Prey Distribution, Physical Habitat Features, and Guild Traits Interact to Produce Contrasting Shorebird Assemblages among Foraging Patches
Source: PLoS One. 2012 Dec 20;7(12):e52694. doi: 10.1371/journal.pone.0052694 (PMC3527609; doi:10.1371/journal.pone.0052694)
Supplement: Table S1 — Total intertidal area (hectares) of each tidal flat at the three tidal stages of observation, as a function of tidal amplitude. (DOCX) [file pone.0052694.s001.docx]

|  |  | Flat | | | | |  |
| --- | --- | --- | --- | --- | --- | --- | --- |
| Tidal Stage | Tidal Range | SE | BR | SH | IS | TC | Observation Dates |
| Mid Ebb Tide | Normal | 0.29 | 0.52 | 0.05 | 0 | 0 | Nov 10, 24-26, 28 |
|  | Spring | 0.29 | 0.52 | 0.05 | 0 | 0 | Dec 13, 15, 16 |
| Late Ebb Tide | Normal | 0.40 | 1.19 | 0.25 | 0 | 0.13 | Nov 10, 24-26, 28 |
|  | Spring | 0.44 | 1.34 | 0.30 | 0.34 | 0.34 | Dec 13, 15, 16 |
| Low Tide | Normal | 0.44 | 1.34 | 0.30 | 0.10 | 0.34 | Oct 27-31, Nov 1, 10, 24-26, 28 |
|  | Spring | 0.54 | 1.53 | 0.50 | 0.74 | 0.80 | Dec 13, 15 |

Flat surface area at the two later tidal stages varied with changing tidal amplitude. Tides on a given observation date were classified as “normal” or “spring” based on aerial exposures recorded in the field: surface areas are calculated for each tidal regime and flat as listed above. SE, Semi-Enclosed Flat; BR, Broad Flat; SH, Shell Flat; IS, Island Flat; TC, Tidal Creek Flat.
