# Supplementary material for: Prey Distribution, Physical Habitat Features, and Guild Traits Interact to Produce Contrasting Shorebird Assemblages among Foraging Patches
Source: PLoS One. 2012 Dec 20;7(12):e52694. doi: 10.1371/journal.pone.0052694 (PMC3527609; doi:10.1371/journal.pone.0052694)
Supplement: Table S2 — Benthic macrofaunal species found in at least five percent of samples. (DOCX) [file pone.0052694.s002.docx]

| Higher taxonomic classification | Species |
| --- | --- |
| Polychaeta | *Aricidea fragilis* |
|  | *Capitella capitata* |
|  | *Eteone* sp. |
|  | *Glycera* sp. |
|  | *Haploscoloplos robustus* |
|  | *Heteromastus filiformis* |
|  | *Nereis* spp. |
|  | *Paraonis* sp. |
|  | Unknown |
| Amphipoda | (All pooled) |
| Bivalvia | *Donax variabilis* |
|  | *Gemma gemma* |
|  | *Mercenaria mercenaria* |
| Gastropoda | *Nassarius obsoletus* |

This benthic species subset was used in place of the complete 67-species list for all benthic-shorebird BEST procedures.
