# Supplementary material for: Prey Distribution, Physical Habitat Features, and Guild Traits Interact to Produce Contrasting Shorebird Assemblages among Foraging Patches
Source: PLoS One. 2012 Dec 20;7(12):e52694. doi: 10.1371/journal.pone.0052694 (PMC3527609; doi:10.1371/journal.pone.0052694)
Supplement: Table S3 — Phylum Polychaeta densities (organisms m−2) by flat. (DOCX) [file pone.0052694.s003.docx]

|  |  | Flat | | | | |
| --- | --- | --- | --- | --- | --- | --- |
| Family | Species | SE | BR | SH | IS | TC |
| Arenicolidae | *Arenicola cristata* | 0 | 2 | 0 | 0 | 0 |
| Capitellidae | *Capitella capitata* | 48.4 | 17.7 | 370.4 | 18.8 | 34.3 |
|  | *Dasybranchus* sp. | 1.9 | 0 | 0 | 0 | 0 |
|  | *Heteromastus filiformis* | 50.3 | 5.9 | 13.6 | 9.4 | 247.7 |
|  | *Notomastus* sp. | 0 | 2 | 0 | 0 | 0 |
|  | Capitellidae- unknown | 34.8 | 0 | 0 | 0 | 0 |
| Cirratulidae | *Tharyx* sp. | 7.7 | 2 | 4.5 | 0 | 15.2 |
| Glyceridae | *Glycera* sp. | 3.9 | 2 | 27.1 | 18.8 | 19.1 |
|  | *Hemipodus roscus* | 0 | 3.9 | 0 | 0 | 0 |
| Goniadidae | *Glycinde solitaria* | 0 | 0 | 4.5 | 0 | 3.8 |
|  | *Goniada* sp. | 0 | 0 | 0 | 9.4 | 0 |
| Lumbrineridae | *Lumbrineris* sp. | 0 | 3.9 | 0 | 0 | 0 |
| Maldanidae | *Clymenella torquata* | 0 | 0 | 18.1 | 28.1 | 0 |
|  | *Rhodine* sp. | 0 | 0 | 0 | 0 | 11.4 |
| Nereididae | *Nereis* spp. | 52.3 | 145.6 | 189.7 | 0 | 11.4 |
| Oenonidae | *Notocirrus spiniferus* | 7.7 | 0 | 0 | 0 | 3.8 |
| Onuphidae | *Onuphis* sp. | 0 | 0 | 4.5 | 0 | 0 |
| Opheliidae | *Armandia agilis* | 1.9 | 0 | 0 | 9.4 | 3.8 |
|  | *Ophelia denticulata* | 3.9 | 2 | 22.6 | 0 | 0 |
|  | Opheliidae- unknown | 1.9 | 0 | 0 | 0 | 0 |
|  | *Travicia* sp. | 0 | 0 | 0 | 0 | 3.8 |
| Orbiniidae | *Haploscoloplos robustus* | 54.2 | 135.7 | 58.7 | 0 | 15.2 |
|  | *Orbinia americana* | 1.9 | 0 | 0 | 0 | 3.8 |
|  | *Scoloplos* sp. | 5.8 | 13.8 | 0 | 0 | 7.6 |
| Paraonidae | *Aricidea fragilis* | 9.7 | 11.8 | 27.1 | 9.4 | 83.8 |
|  | *Paraonis* sp. | 9.7 | 3.9 | 176.2 | 28.1 | 30.5 |
| Pectinariidae | *Pectinaria* *gouldi* | 0 | 2 | 0 | 0 | 3.8 |
| Phyllodocidae | *Eteone* sp. | 1.9 | 9.8 | 18.1 | 0 | 0 |
|  | *Paranaitis* sp. | 0 | 2 | 0 | 0 | 3.8 |
|  | *Phyllodoce* sp. | 0 | 0 | 0 | 9.4 | 3.8 |
| Spionidae | *Polydora* sp. | 0 | 0 | 13.6 | 0 | 0 |
|  | Spionidae- unknown | 0 | 0 | 4.5 | 0 | 0 |
| Unknown | unidentified polychaetes | 48.4 | 15.7 | 18.1 | 18.8 | 26.7 |

Flat abbreviations are as in Table S1.
