# Supplementary material for: Prey Distribution, Physical Habitat Features, and Guild Traits Interact to Produce Contrasting Shorebird Assemblages among Foraging Patches
Source: PLoS One. 2012 Dec 20;7(12):e52694. doi: 10.1371/journal.pone.0052694 (PMC3527609; doi:10.1371/journal.pone.0052694)
Supplement: Table S4 — Phylum Arthropoda densities (organisms m−2) by flat. (DOCX) [file pone.0052694.s004.docx]

|  |  |  | Flat | | | | |
| --- | --- | --- | --- | --- | --- | --- | --- |
| ITC | Family | Species | SE | BR | SH | IS | TC |
| Amphipoda | … | Amphipoda- all | 211 | 464.2 | 289.1 | 65.7 | 122 |
| Caprellidae | … | Caprellidae- unknown | 0 | 0 | 0 | 0 | 30.5 |
| Decapoda | Grapsidae | *Sesarma reticulatum* | 0 | 0 | 4.5 | 0 | 0 |
|  | Ocypodidae | *Uca pugilator* | 7.7 | 0 | 0 | 0 | 0 |
|  | Pinnotheridae | *Pinnixa* sp. | 0 | 5.9 | 0 | 0 | 19.1 |
|  | … | Brachyura- unknown | 1.9 | 0 | 0 | 9.4 | 0 |
|  | … | Paguroidea- unknown | 0 | 2 | 4.5 | 18.8 | 0 |
| Isopoda | Anthuridae | *Cyathura polita* | 0 | 2 | 0 | 0 | 0 |
|  | Sphaeromatidae | *Sphaeroma quadridentatum* | 0 | 2 | 4.5 | 0 | 0 |
|  | … | Isopoda- unknown | 0 | 2 | 0 | 0 | 0 |

ITC, Intermediate taxonomic classification; flat abbreviations are as in Table S1. Ellipsis indicates unknown classification.
