# Supplementary material for: Prey Distribution, Physical Habitat Features, and Guild Traits Interact to Produce Contrasting Shorebird Assemblages among Foraging Patches
Source: PLoS One. 2012 Dec 20;7(12):e52694. doi: 10.1371/journal.pone.0052694 (PMC3527609; doi:10.1371/journal.pone.0052694)
Supplement: Table S5 — Phylum Mollusca densities (organisms m−2) by flat. (DOCX) [file pone.0052694.s005.docx]

|  |  |  | Flat | | | | |
| --- | --- | --- | --- | --- | --- | --- | --- |
| ITC | Family | Species | SE | BR | SH | IS | TC |
| Bivalvia | Donacidae | *Donax variabilis* | 1.9 | 43.3 | 18.1 | 37.5 | 22.9 |
|  | Mactridae | *Spisula solidissima* | 3.9 | 0 | 0 | 0 | 0 |
|  | Mytilidae | *Brachidontes exustus* | 0 | 0 | 9 | 0 | 0 |
|  | Semelidae | *Semele proficua* | 1.9 | 0 | 0 | 0 | 0 |
|  | Solecurtidae | *Tagelus* spp. | 0 | 2 | 4.5 | 0 | 15.2 |
|  | Tellinidae | *Tellina* spp. | 1.9 | 2 | 0 | 0 | 3.8 |
|  | Veneridae | *Chione* sp. | 0 | 2 | 4.5 | 0 | 0 |
|  |  | *Dosinia* spp. | 0 | 0 | 0 | 0 | 7.6 |
|  |  | *Gemma gemma* | 42.6 | 2 | 0 | 0 | 3.8 |
|  |  | *Mercenaria mercenaria* | 9.7 | 7.9 | 0 | 0 | 11.4 |
| Gastropoda | Ellobiidae | *Melampus bidentatus* | 0 | 0 | 0 | 18.8 | 26.7 |
|  | Littorinidae | *Littorina irrorata* | 54.2 | 0 | 0 | 0 | 0 |
|  | Nassariidae | *Nassarius obsoletus* | 58.1 | 0 | 0 | 0 | 15.2 |
|  |  | *Nassarius vibex* | 0 | 0 | 9 | 0 | 0 |
|  | Naticidae | *Neverita duplicata* | 0 | 2 | 0 | 0 | 0 |

ITC, Intermediate taxonomic classification; flat abbreviations are as in Table S1
