# Supplementary material for: Prey Distribution, Physical Habitat Features, and Guild Traits Interact to Produce Contrasting Shorebird Assemblages among Foraging Patches
Source: PLoS One. 2012 Dec 20;7(12):e52694. doi: 10.1371/journal.pone.0052694 (PMC3527609; doi:10.1371/journal.pone.0052694)
Supplement: Table S6 — Flat-specific densities (organisms m−2) of organisms from additional phyla. (DOCX) [file pone.0052694.s006.docx]

|  |  |  |  | Flat | | | | |
| --- | --- | --- | --- | --- | --- | --- | --- | --- |
| Phylum | ITC | Family | Species | SE | BR | SH | IS | TC |
| Hemichordata | Enteropneusta | Harrimaniidae | *Saccoglossus kowalevskii* | 0 | 3.9 | 0 | 9.4 | 7.6 |
|  |  | … | Enteropneusta- unknown | 0 | 2 | 0 | 0 | 0 |
| Nemertea | Anopla | Lineidae | *Micrura leidyi* | 0 | 0 | 0 | 9.4 | 0 |
|  | … | … | Nemertea- unknown | 1.9 | 5.9 | 4.5 | 28.1 | 0 |
| Nematoda | … | … | Nematoda- unknown | 0 | 2 | 0 | 0 | 30.5 |
| Cnidaria | Anthozoa | Edwardsiidae | *Edwardsia elegans* | 0 | 0 | 0 | 9.4 | 0 |
| Platyhelminthes | Turbellaria | Stylochidae | *Coronadena mutabilis* | 0 | 0 | 0 | 0 | 3.8 |
| Echinodermata | Holothuroidea | … | Holothuroidea- unknown | 0 | 2 | 0 | 0 | 0 |

ITC, Intermediate taxonomic classification; flat abbreviations are as in Table S1. Ellipsis indicates unknown classification.
