# Supplementary material for: Prey Distribution, Physical Habitat Features, and Guild Traits Interact to Produce Contrasting Shorebird Assemblages among Foraging Patches
Source: PLoS One. 2012 Dec 20;7(12):e52694. doi: 10.1371/journal.pone.0052694 (PMC3527609; doi:10.1371/journal.pone.0052694)
Supplement: Table S7 — Results of one-way ANOVAs comparing benthic invertebrate abundance among tidal flats at each tidal stage, and post hoc contrasts using Tukey-Kramer HSD. (DOCX) [file pone.0052694.s007.docx]

| Taxon | Statistic | Tidal stage | | |
| --- | --- | --- | --- | --- |
|  |  | Mid Ebb^a^ | Late Ebb^b^ | Low^c^ |
| Polychaetes | F ratio | 3.4 | 3.99 | 6.69 |
|  | P-value | 0.041 | 0.012 | < 0.001 |
|  | TK post hoc | SH>SE | SH>BR | SH=TC=SE>IS |
| Crustaceans | F ratio | … | 8.04 | 3.18 |
|  | P-value | … | <0.001 | 0.018 |
|  | TK post hoc | … | BR>SH=SE | NSD |
| Bivalves | F ratio | … | … | … |
|  | P-value | … | … | … |
|  | TK post hoc | … | … | … |
| Gastropods | F ratio | … | … | 2.7 |
|  | P-value | … | … | 0.037 |
|  | TK post hoc | … | … | SE>BR |

Only significant ANOVA results are reported (α = 0.05); ellipsis indicates non-significant results. Only statistically significant HSD results are listed. NSD, no significant differences. All five flats were included in these analyses: BR, Broad Flat; SE, Semi-Enclosed Flat; SH, Shell Flat; TC, Tidal Creek Flat; IS, Island Flat.

^a^ Flats compared: BR, SE, SH; degrees of freedom: 2, 48

^b^ Flats compared: BR, SE, SH, TC; df: 3, 62

^c^ Flats compared: BR, SE, SH, TC, IS; df: 4,75
