# Supplementary material for: Prey Distribution, Physical Habitat Features, and Guild Traits Interact to Produce Contrasting Shorebird Assemblages among Foraging Patches
Source: PLoS One. 2012 Dec 20;7(12):e52694. doi: 10.1371/journal.pone.0052694 (PMC3527609; doi:10.1371/journal.pone.0052694)
Supplement: Table S8 — Results of one-way ANOVAs comparing benthic invertebrate biomass among tidal flats at each tidal stage, and post hoc contrasts using Tukey-Kramer HSD. (DOCX) [file pone.0052694.s008.docx]

|  |  | Tidal stage | | |
| --- | --- | --- | --- | --- |
| Taxon | Statistic | Mid Ebb | Late Ebb | Low |
| Polychaetes | F ratio | 4.9 | … | … |
|  | P-value | 0.012 | … | … |
|  | TK post hoc | SH>BR=SE | … | … |
| Crustaceans | F ratio | … | 2.77 | … |
|  | P-value | … | 0.049 | … |
|  | TK post hoc | … | NSD | … |
| Bivalves | F ratio | … | … | 3.92 |
|  | P-value | … | … | 0.006 |
|  | TK post hoc | … | … | TC>BR=SE=IS |
| Gastropods | F ratio | … | 2.96 | 2.54 |
|  | P-value | … | 0.039 | 0.047 |
|  | TK post hoc | … | SE>BR | NSD |

Only significant ANOVA results are reported (α = 0.05); ellipsis indicates non-significant results. Only statistically significant HSD results are listed. See Table S7 for abbreviations, degrees of freedom, and flats compared at each tidal stage.
