# Supplementary material for: Prey Distribution, Physical Habitat Features, and Guild Traits Interact to Produce Contrasting Shorebird Assemblages among Foraging Patches
Source: PLoS One. 2012 Dec 20;7(12):e52694. doi: 10.1371/journal.pone.0052694 (PMC3527609; doi:10.1371/journal.pone.0052694)
Supplement: Table S9 — Results of post hoc pairwise comparisions of flats following Analysis of Similarity (ANOSIM) on benthic invertebrate community composition dataset of abundances by species (or genus, and in some cases higher taxon). (DOCX) [file pone.0052694.s009.docx]

| Flat pairs | R statistic | p |
| --- | --- | --- |
| SE — BR | 0.129 | 0.001 |
| SE — SH | 0.023 | 0.255 |
| SE — IS | 0.191 | 0.001 |
| SE — TC | 0.033 | 0.102 |
| BR — SH | 0.202 | 0.001 |
| BR — IS | 0.363 | 0.001 |
| BR — TC | 0.243 | 0.001 |
| SH — IS | 0.381 | 0.001 |
| SH — TC | 0.316 | 0.001 |
| IS — TC | 0.285 | 0.001 |

Flat abbreviations are as in Table S7.
