# Supplementary material for: Prey Distribution, Physical Habitat Features, and Guild Traits Interact to Produce Contrasting Shorebird Assemblages among Foraging Patches
Source: PLoS One. 2012 Dec 20;7(12):e52694. doi: 10.1371/journal.pone.0052694 (PMC3527609; doi:10.1371/journal.pone.0052694)
Supplement: Table S10 — Results of Tukey-Kramer HSD post hoc comparisons of all possible pairs of flats based on percentage composition of each size class of sediments. (DOCX) [file pone.0052694.s010.docx]

| Grain size class | Post hoc results |
| --- | --- |
| Silt/clay | TC >SE > SH; TC >IS=BR |
| Very fine sand | IS > BR=SH=TC >SE |
| Fine sand | BR=IS=TC=SE >SH |
| Medium sand | SE > BR=TC > IS; SE >SH |
| Coarse/ very coarse sand | SH > SE=BR=TC=IS |
| Gravel | SH >IS=BR=SE=TC |

Post hoc comparisons were conducted after one-way ANOVAs revealed statistically significant variation among flats for each sediment size class. Only significant (α = 0.05) pairwise contrasts are listed. Flat abbreviations are as in Table S7.
